# Physiological Roles of Calpain 1 Associated to Multiprotein NMDA Receptor Complex

**DOI:** 10.1371/journal.pone.0139750

**Published:** 2015-10-02

**Authors:** Monica Averna, Matteo Pellegrini, Chiara Cervetto, Marco Pedrazzi, Margherita Bavestrello, Roberta De Tullio, Franca Salamino, Sandro Pontremoli, Edon Melloni

**Affiliations:** 1 Department of Experimental Medicine (DIMES)—Biochemistry Section, University of Genova, Viale Benedetto XV, 1–16132, Genova, Italy; 2 Center of Excellence for Biomedical Research (CEBR), University of Genova, Viale Benedetto XV, 1–16132, Genova, Italy; 3 Department of Pharmacy, Pharmacology and Toxicology Section, University of Genova, Viale Cembrano 4, 16148, Genova, Italy; University of Louisville, UNITED STATES

## Abstract

We have recently demonstrated that in resting conditions calpain 1, but not calpain 2, is specifically associated to the N-Methyl-D-Aspartate receptor (NMDAR) multiprotein complex. We are here reporting that in SKNBE neuroblastoma cells or in freshly isolated nerve terminals from adult rat hippocampus, the proteolytic activity of calpain 1 resident at the NMDAR is very low under basal conditions and greatly increases following NMDAR stimulation. Since the protease resides at the NMDAR in saturating amounts, variations in Ca^2+^ influx promote an increase in calpain 1 activity without affecting the amount of the protease originally associated to NMDAR. In all the conditions examined, resident calpain 1 specifically cleaves NR2B at the C-terminal region, leading to its internalization together with NR1 subunit. While in basal conditions intracellular membranes include small amounts of NMDAR containing the calpain-digested NR2B, upon NMDAR stimulation nearly all the receptor molecules are internalized. We here propose that resident calpain 1 is involved in NMDAR turnover, and following an increase in Ca^2+^ influx, the activated protease, by promoting the removal of NMDAR from the plasma membranes, can decrease Ca^2+^ entrance through this channel. Due to the absence of calpastatin in such cluster, the activity of resident calpain 1 may be under the control of HSP90, whose levels are directly related to the activation of this protease. Observations of different HSP90/calpain 1 ratios in different ultrasynaptic compartments support this conclusion.

## Introduction

Multiple lines of evidence indicate that the Ca^2+^-dependent protease calpain plays critical roles in both physiological and pathological conditions [1–6]. In particular, in brain tissue, calpain activation is normally mediated by Ca^2+^ entry via stimulation of NMDAR, and whose NR2A and NR2B subunits are both calpain substrates [7–10]. Moreover, it is well known that following receptor stimulation, these NMDAR subunits, involved in the anchoring of NMDAR to the cytoskeleton by protein-protein interactions [11–22], participate to the activation of downstream intracellular signaling pathways.

The direct functional correlation between calpain and NMDAR has been extensively investigated and the data obtained have often suggested different conclusions. In particular the major controversial problem concerns the hypothesis that calpain activation, induced by extensive stimulation of NMDAR, is directly responsible for excitotoxicity and cell death [23–28]. A second issue concerns the fate of NMDAR after the digestion of NR2 subunits [9, 29]. Furthermore, a number of studies have proposed that calpain-mediated degradation of NMDAR could act as an initial defense mechanism by reducing the number of functional NMDAR molecules, and consequently decreasing the extent of Ca^2+^ influx [30–32]. More recently it has been reported that the activation of calpain 1 coupled with synaptic NMDAR can exert a role in neuroprotection, whereas the activation of calpain 2 coupled with extrasynaptic NMDAR is involved in neurodegeneration [33]. From our recent observations [34] an additional contribution to the understanding of the mechanism and the biological role of the proteolytic processing of NMDAR emerges. Our data indicate that, in SKNBE neuroblastoma cells, the chaperone HSP90 plays a specific role in the control of the dynamic activation of calpain 1 and that the chaperone can assist the protease during its recruitment in the multiprotein NMDAR cluster [34]. These observations are consistent with the finding that calpain 1, but not calpain 2, resides in small but saturating amounts at the NMDAR complex together with a large excess of HSP90 [34].

To obtain additional information on the mechanism and the physiological significance of the relationship between calpain 1 and NMDAR, we have investigated the role of resident calpain 1 in the proteolytic processing of NMDAR.

We here demonstrate that, in human neuroblastoma SKNBE cells, the NR2B subunit of NMDAR, predominantly expressed in these cells [35, 36], undergoes a selective “in situ” proteolytic digestion at the C-terminal region, by resident calpain 1. We observed that NR2B proteolysis normally occurs at limited extent in resting cells and at a much higher rate following cell stimulation with non-toxic NMDA concentrations. In both conditions NMDAR, containing the digested NR2B subunit, undergoes internalization into membrane vesicles.

On the basis of these observations, we are now proposing that in basal conditions calpain 1 resident in the NMDAR complex is involved in the normal turnover of the receptor. Following NMDAR stimulation, an increase in the proteolytic processing of NR2B is observed. As a consequence, an almost complete internalization of the digested receptor occurs. This process may represent in SKNBE cells an initial defense mechanism that operates by reducing NMDAR-mediated Ca^2+^ influx.

These observations are further strengthened by the fact that also in synaptosomes and their ultrastructural fractions prepared from adult rat hippocampus, calpain 1 is in a resident form and importantly is responsible for similar proteolytic processing of NMDAR. Purified synaptosomes, prepared from rodent mature central nervous system, and characterized in our laboratories [37, 38, 39], ensure negligible contamination by non-neuronal cells, like atrocytes, microglia or oligodendrocytes [40, 41] and can provide a helpful model that mirrors the in vivo NMDAR subunit composition. The results obtained show that the rate of calpain-mediated digestion of NR2B is inversely correlated to the availability of the chaperone HSP90 inserted in the NMDAR cluster.

## Materials and Methods

### Reagents and Antibodies

Leupeptin, aprotinin, Pefabloc^®^ SC, Tween^®^ 20, Triton^®^ X-100, C.I-2 (calpain inhibitor 2), glycine and neutral red solution all the salts were purchased from Sigma-Aldrich. ECL ADVANCE® Detection System and Protein G-Sepharose were obtained from GE Healthcare. t-BOC (t-butoxycarbonyl)-Leu-Met-CMAC (7-amino-4-chlorometylcoumarin), a fluorogenic calpain substrate was purchased from Molecular Probes (Invitrogen). Sodium deoxycholate was from Merck (Milan, Italy). Anti-NMDAR2B monoclonal antibody (13/NMDAR2B) and anti-HSP90 monoclonal antibody (68/HSP90) were purchased from BD Transduction Laboratories TM. Anti-NR1, CT monoclonal antibody and anti-synaptophysin polyclonal antibody were purchased from Millipore. Monoclonal anti-calpain 1 (calpain I, subunit p80) clone 15C10 and monoclonal anti-calpain 2 (Domain III/IV) clone 107–82 were obtained from Sigma-Aldrich. Calpastatin was detected with the monoclonal anti-calpastatin (Domain IV) clone 1F7E3D10 purchased from Calbiochem. Anti-syntaxin monoclonal antibody (4H256) was purchased from GeneTex. Anti-PSD95 polyclonal antibody was obtained from Cell Signaling.

### Animals

Adult male rats (Sprague Dawley 200–250 g) were housed at constant temperature (22±1°C) and relative humidity (50%) under a regular light-dark schedule (lights on 7 AM-7 PM). Food and water were freely available. The experimental procedures and animal care complied with the European Communities Parliament and Council Directive of 22 September 2010 (2010/63/EU) and with the Italian D.L. n. 26/2014, and were approved by the Italian Ministry of Health in accordance with Decreto Ministeriale 116/1992 (protocol number 26768 of November 2012). Any effort was made to minimize the number of animals used and their suffering.

### Preparation of purified nerve terminals

Synaptosomes were prepared as previously described [35, 39] according to [42]. After decapitation the hippocampus was rapidly removed; the tissue was placed in ice-cold medium (0.32 M sucrose, buffered at pH 7.4 with Tris-HCl). Briefly, the tissue was homogenized in 10 volumes of Tris-buffered sucrose, using a glass-Teflon tissue grinder (clearance 0.25 mm). The homogenate was centrifuged (1,000 g at 4°C for 5 min) to remove nuclei and debris, and the supernatant was gently stratified on a discontinuous Percoll gradient (2%, 6%, 10%, and 20% v/v in Tris-buffered sucrose) and centrifuged at 33,500 g for 5 min. The layer between 10% and 20% Percoll was collected and washed by centrifugation. Synaptosomes obtained from rat brain were proven positive for the neuronal markers synaptophysin and MAP-2, and negative for the glial, oligodendrocyte or microglial markers GFAP, RIP or integrin-α-M, indicating negligible contamination by non-neuronal cells [39, 40]. The synaptosomal fraction obtained from rodent hippocampus has been previously proven positive for the neuronal markers MAP-2 and only scantily contaminated by the glial markers GFAP; biochemical and functional data both supported that the synaptosomal fraction was largely free from astrocyte processes or non-neuronal cells [41].

Synaptosomal pellets were suspended in HEPES standard medium with the following composition (mM): NaCl 128, KCl 2.4, MgSO_4_ 1.2, CaCl_2_ 1.2, KH_2_PO_4_ 1.2, HEPES 10, glucose 10 (pH 7.3–7.4).

### Cell culture

SKNBE human neuroblastoma cells (Interlab Cell Line Collection ICLC, HTL96015, Italy) were maintained in continuous culture at 37°C (5% CO_2_) with Dulbecco’s minimal essential medium (DMEM) supplemented with 10% fetal bovine serum, 10 U/mL penicillin, 100 mg/mL streptomycin and 2 mM L-glutamine.

### Assay of intracellular calpain activity

SKNBE cells were incubated, at 37°C for 20 min, in 100 μL of oxygenated 10 mM HEPES, 0.14 M NaCl, 5 mM KCl, 5 mM glucose, 1 mM MgCl_2_, pH 7.4 (HEPES buffer) containing 50 μM t-Boc-Leu-Met-CMAC fluorogenic calpain substrate in 96-well microplate. Cells were then washed twice with HEPES buffer to remove substrate excess and without or after addition of 0.1 mM NMDA in 100 μL of HEPES buffer, containing 10 μM glycine, and 1 mM CaCl_2_, the fluorescence emission was continuously monitored for 2 hours with a Mithras LB 940 plate reader (Berthold Technologies). The excitation/emission wavelengths were 355/485 nm, respectively.

### [Ca^2+^]_i_ Assay

2×10^4^ cells were incubated in 200 μl of HEPES buffer containing 10 μM Calcium Green™-1, AM. After 40 min at 37°C cells were washed with HEPES buffer and NMDA was added in 100 μl of HEPES buffer. The fluorescence intensity (Excitation 485 nm; Emission 535 nm) was measured before (F0) and 5 min after the addition of stimuli (F) using the top reading mode in the fluorescence multilabel reader LB 940 Mithras (Berthold Italia). Variations of the fluorescence values were calculated as the difference between F and F0.

### Confocal microscopy imaging and fluorescence quantification

SKNBE cells (10^5^) were fixed and permeabilized by the Triton/paraformaldehyde method, as described in [43]. Cells were incubated with 10 μg/mL anti-NR1 or anti-NMDAR2B monoclonal antibodies diluted in PBS solution, containing 5% (v/v) FBS. After 3 h incubation at 25°C, cells were washed three times with PBS solution and exposed for 1 h to 4 μg/mL chicken anti-mouse IgG Alexa fluor® 488-conjugate secondary antibody, Molecular Probes. Images were collected using a Bio-Rad MRC1024 confocal microscopy, with a 60× Plan Apo objective with numerical aperture 1.4. The fluorescence intensity in each image was quantified using LaserPix software (Bio-Rad) following the procedure described in [44].

### Cell viability assays

Cell viability was measured by means of the neutral red uptake [45].

### Immunoprecipitation and Immunoblotting

SKNBE cells (10^7^) and synaptosomes were lysed in 50 mM sodium borate buffer, 1 mM EDTA, 10 μg/mL aprotinin, 100 μg/mL leupeptin, 2 mM Pefabloc^®^ SC, pH 7.5 by three freeze-thaw cycles and briefly sonicated. Membrane fractions of cells and synaptosomes were obtained by centrifugation at 100,000 g for 15 min at 4°C, washed in 50 mM sodium borate buffer, 0.1 mM EDTA, pH 7.5 (total membrane fraction), and solubilized in 50 mM sodium borate buffer, 0.1 mM EDTA, 1% sodium deoxycholate, pH 9.0 at 37°C for 60 min. After centrifugation at 100,000 g for 30 min at 4°C, the pH was adjusted to pH 8.0, and Triton X-100 was added to a final concentration of 0.1%. The detergent-soluble portion was dialyzed against 50 mM sodium borate buffer, 0.1 mM EDTA, 0.1% Triton X-100, pH 7.5 (IP buffer), by diafiltration using centrifugal filter devices (10 kDa cut-off) Amicon Ultra-4 (Millipore). Before immunoprecipitation procedure, samples (500 μg) were pre-cleared for 60 min at 4°C using 30 μL of Protein G-sepharose diluted 1:1 with IP buffer. Immunoprecipitation was carried out using 1 μg of anti-NR1 antibody. The immunoprecipitated material was eluted with 30 μL of SDS-PAGE loading buffer [46], heated for 5 min at 95°C, and submitted to 8% SDS-PAGE. Proteins were separated by 8% SDS-PAGE, blotted [47] onto a nitrocellulose membrane (Bio-Rad) and probed with the specific mAbs. The immunoreactive bands were developed with an ECL detection system, detected with a Bio-Rad Chemi Doc XRS apparatus, and quantified using the Quantity One software, release 4.6.1 (Bio-Rad).

### Assay of calpain activity in NR1-immunoprecipitates

NR1-immunoprecipitation was performed using SKNBE cells (10^5^) as described above in this section and the immunoprecipitated material, suspended in 100 μL of HEPES buffer containing 5 μM or 1 mM CaCl_2_, was incubated at 37°C for 10 min in the presence of 50 μM t-Boc-Leu-Met-CMAC fluorogenic calpain substrate in 96-well microplate. The fluorescence emission was evaluated with a Mithras LB 940 plate reader (Berthold Technologies) at the excitation/emission wavelengths 355/485 nm, respectively.

### Isolation of the Plasma and Intracellular Membranes from SKNBE cells

Total membrane fraction (100 μg in 0.5 mL of 50 mM sodium borate buffer, 0.1 mM EDTA, pH 7.5) prepared from SKNBE cells as described above was fractionated on a discontinuous sucrose density gradient [10, 20, 30 and 50% (w/v)]. After centrifugation at 38,000 rpm in a Beckman Coulter TLA-100.4 rotor for 30 min, membranes were recovered into two fractions layered at 10% and 30% sucrose interface. They were separately collected, suspended in 0.2 mL of sodium borate 50 mM, pH 7.5 containing 1 mM EDTA and 5′-nucleotidase activity was assayed [48], as plasma membranes marker. Aliquots (30 μL) of the same samples were suspended in SDS-PAGE loading buffer [46], heated for 5 min at 95°C, and submitted to 8% SDS-PAGE. Proteins were then blotted and probed with the specific mAbs.

### Biotinylation

Confluent (90–95%) SKNBE cells grown on four T75 cm^2^ flasks were incubated for 2 hours at 37°C in HEPES buffer in the absence or presence of 0.1 mM NMDA. Alternatively, synaptosomes were incubated for 12 min at 37°C in HEPES buffer in the absence or presence of 0.1 mM NMDA. After incubation cells and synaptosomes were washed twice with ice-cold PBS and cell surface biotinylation using EZ-LinkHSulfo-NHS-SS-biotin was performed with the Pierce cell surface protein isolation kit according to the manufacturer’s instructions (Thermo Scientific). In brief, cells and synaptosomes were biotinylated and lysed in 500 μL of lysis buffer supplemented with protease inhibitor cocktail (1:100, Sigma-Aldrich) and 1 mM AEBSF. For each experimental condition, the same amount of protein (500 μg) was incubated with 500 μL of NeutrAvidin beads for 1 h at room temperature and, after collecting unbound proteins (not biotinylated), was eluted with 500 μL of SDS sample buffer (62.5 mM Tris-HCl, pH 6.8, 1% SDS, 10% glycerol, and 50 mM DTT). Aliquots (30 μL) of biotinylated and not biotinylated proteins were heated for 5 min at 95°C, and submitted to 8% SDS-PAGE. Proteins were then blotted and probed with the specific mAbs.

### Ultrastructural fractionation

The ultrastructural fractionation of synaptosomes was performed according to [49]. The synaptosome pellets suspended in 150 μL of HEPES buffer were diluted 1:10 in ice-cold 0.1 mM CaCl_2_ and mixed with an equal volume of 2% Triton X-100, 40 mM Tris, pH 6.0 in the presence of protease inhibitors. Following 30 min of incubation at 4°C the insoluble material (synaptic junctions) was pelletted by centrifugation (40,000 g for 30 min, 4°C). The supernatant (non synaptic fraction) was decanted and proteins precipitated with 4 volumes of acetone at -20°C overnight. The proteins were centrifuged (13,700 g for 30 min, -20°C). The synaptic junction pellet was suspended in 10 volumes of 1% Triton X-100, 20 mM Tris, pH 8.0 and incubated for 30 min at 4°C and then centrifuged (40,000 g for 30 min, 4°C). The pellet contained the insoluble postsynaptic density and the supernatant contained the presynaptic compartment. The proteins in the supernatant were acetone precipitated as described above. The pellets of the three ultrasynaptic fractions were suspended in 500 μL of 50 mM sodium borate buffer, 0.1 mM EDTA, 1% sodium deoxycholate, pH 9.0 at 37°C for 60 min and the detergent soluble proteins were treated for immunoprecipitation as described above.

Before the immunoprecipitation procedure, aliquots (20 μg, determined following Lowry method) of the samples were suspended in SDS-PAGE loading buffer and submitted to 10% SDS-PAGE. Proteins were then blotted and probed with the specific mAbs for the biochemical characterization of the fractions.

### Statistical analysis

Data are presented as mean ± SEM. Significance of the difference was analyzed by *t*-test or by ANOVA followed by post-hoc Tukey’s test, using the Prism 5.0 software package (GraphPad Software), with statistical significance taken at p < 0.05.

## Results

### Calpain 1 associated to NMDAR is active in basal conditions

We have previously demonstrated that in human neuroblastoma SKNBE cells calpain 1, together with its modulator HSP90, is constitutively associated to NR1/NR2B protein cluster [34].

**Fig 1:**
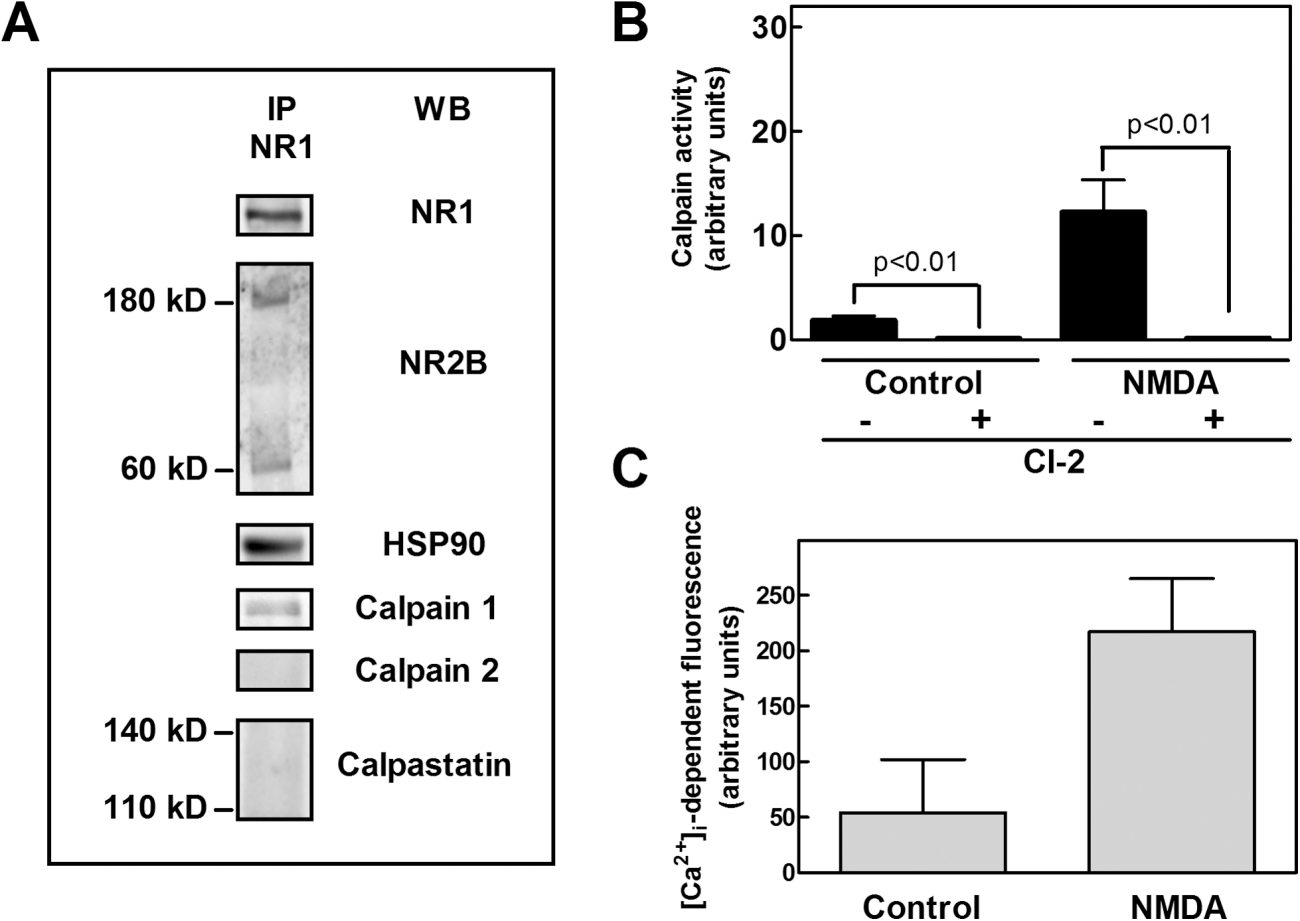
NMDAR cluster and calpain activity in SKNBE cells. (A) SKNBE cells were lysed to perform immunoprecipitation with 1 μg of anti-NR1 antibody. The immunoprecipitated material (IP NR-1) was analyzed by immunoblotting (WB) to detect the indicated proteins. Each immunoblot is representative of four different experiments. (B) SKNBE cells (2 × 10^5^) were incubated in 100 μL of isotonic HEPES buffer containing 50 μM t-Boc-Leu-Met-CMAC fluorogenic calpain substrate (see Methods). Cells were then washed and suspended in 100 μL of HEPES buffer containing 10 μM glycine and 1 mM CaCl_2_. Calpain activity was measured in the absence (Control) or presence (NMDA) of 100 μM NMDA. Calpain activity was also measured in cells pre-incubated for 30 minutes with 1 μM Calpain inhibitor 2 (CI-2). The values reported are the arithmetical mean ± SEM of four different experiments, and *p* values were calculated according to *t*-test. (C) Calcium Green™-loaded cells were exposed to the indicated stimuli. Data are means ± SEM from two independent experiments in duplicate.

To define the role of this resident calpain, we have first explored if the protease, in its associated form, expresses the catalytic activity in unstimulated SKNBE cells. As shown in Fig 1A, the immunoprecipitation with an anti-NR1 antibody revealed that, in addition to the native 180 kD NR2B subunit, a second 60 kD NR2B band was present in NMDAR cluster. This low molecular weight NR2B was similar to the fragment generated by calpain-mediated selective cleavage of NR2B at the C-terminal region [10]. The presence of this fragment suggests not only that calpain 1 is specifically and constitutively associated to NMDAR receptor, but also that the protease is partially active, even in the absence of specific cell stimulation. As we have reported previously [34], HSP90 is also present in this cluster, whereas both calpain 2 and calpastatin are not detectable. Thus, the association of calpain 1 to NMDAR is highly specific.

To verify if the calpain 1 at the NMDAR is responsible for the presence of digested NR2B, we first loaded unstimulated SKNBE cells with a fluorescent calpain substrate and assayed the protease activity. As shown in Fig 1B, in basal conditions a low calpain activity was detectable and the proteolysis was completely blocked by the synthetic calpain inhibitor-2 (CI-2) [50]. Then, cells were exposed to 100 μM NMDA, a condition promoting an influx of Ca^2+^ ions (Fig 1C) sufficient to activate calpain without affecting cell viability (S1 Fig). Indeed, less than 2% of cell death was detectable after 24 hours of cell incubation with 100 μM NMDA. In this condition the protease activity increased 5−6-fold as compared to that detected in unstimulated cells, and was still fully inhibited by CI-2 treatment.

**Fig 2:**
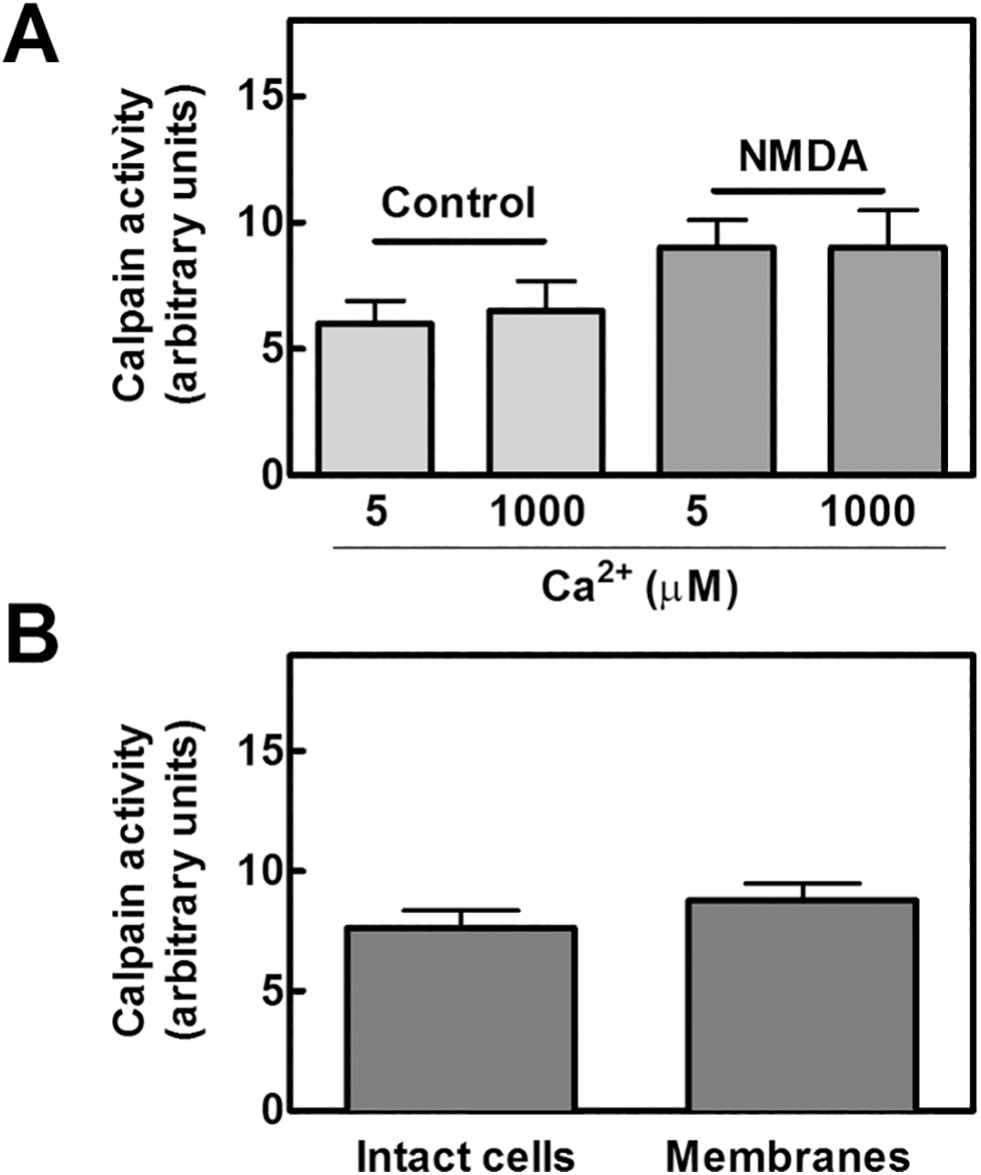
Calpain activity in NR1-immunoprecipitates from SKNBE cells. (A) SKNBE cells (10^5^) untreated (Control) or treated with 100 μM NMDA and 10 μM glycine for 2 hours (NMDA) were lysed to perform immunoprecipitation with 1 μg of anti-NR1 antibody. The immunoprecipitated material was suspended in 100 μL of HEPES buffer and, after addition of 5 or 1000 μM CaCl_2_, incubated at 37°C for 10 min in the presence of 50 μM t-Boc-Leu-Met-CMAC fluorogenic calpain substrate. Calpain activity was measured as described in Methods and the values are reported as difference between the fluorescence monitored at the indicated calcium concentration minus the one monitored in 1 mM EDTA. The values reported are the arithmetical mean ± SEM of four different experiments. (B) Calpain activity was assayed in NR1-immunoprecipitates from 10^5^ SKNBE cells treated as in (A) (Membranes). Intracellular calpain activity was also measured in 10^5^ SKNBE cells treated with 100 μM NMDA and 10 μM glycine for 2 hours (Intact cells).

To establish if this proteolytic activity could be directly attributed to NMDAR resident calpain 1, NR1 and NR2B subunits were immunoprecipitated from untreated and NMDA-treated cells, and calpain activity was then assayed in the immunoprecipitated material. The activity was measured in the presence of 5 μM or 1 mM Ca^2+^, in order to quantify the level of the protease and to identify the isoform associated to the NMDAR complex. As shown in Fig 2A, we detected in both Ca^2+^ concentrations, appreciable and comparable amounts of calpain activity. The fact that, all the activity is already measurable at the lower calcium concentration, indicates that only calpain 1 is present in the NMDAR complex. Moreover, since NMDA stimulation did not induce an increase in the total calpain activity associated to NMDAR (see Fig 2A), it can be concluded that the amount of this resident calpain 1 is not dependent on a Ca^2+^-mediated translocation process, and that the association to the NMDAR complex occurs in a saturating amount already in the absence of stimuli. Finally, as shown in Fig 2B, since calpain activity detected in intact NMDA-stimulated cells is similar to that measured at the NMDAR, it can be concluded that the proteolysis detected is exclusively catalyzed by calpain 1 resident at the NMDAR cluster. Taken together these observations indicate that the proteolytic activity of resident calpain 1 is responsible for the constitutive digestion of NR2B subunits that yields the 60 kD NR2B fragment.

### Resident calpain 1 participates to NMDAR turnover

**Fig 3:**
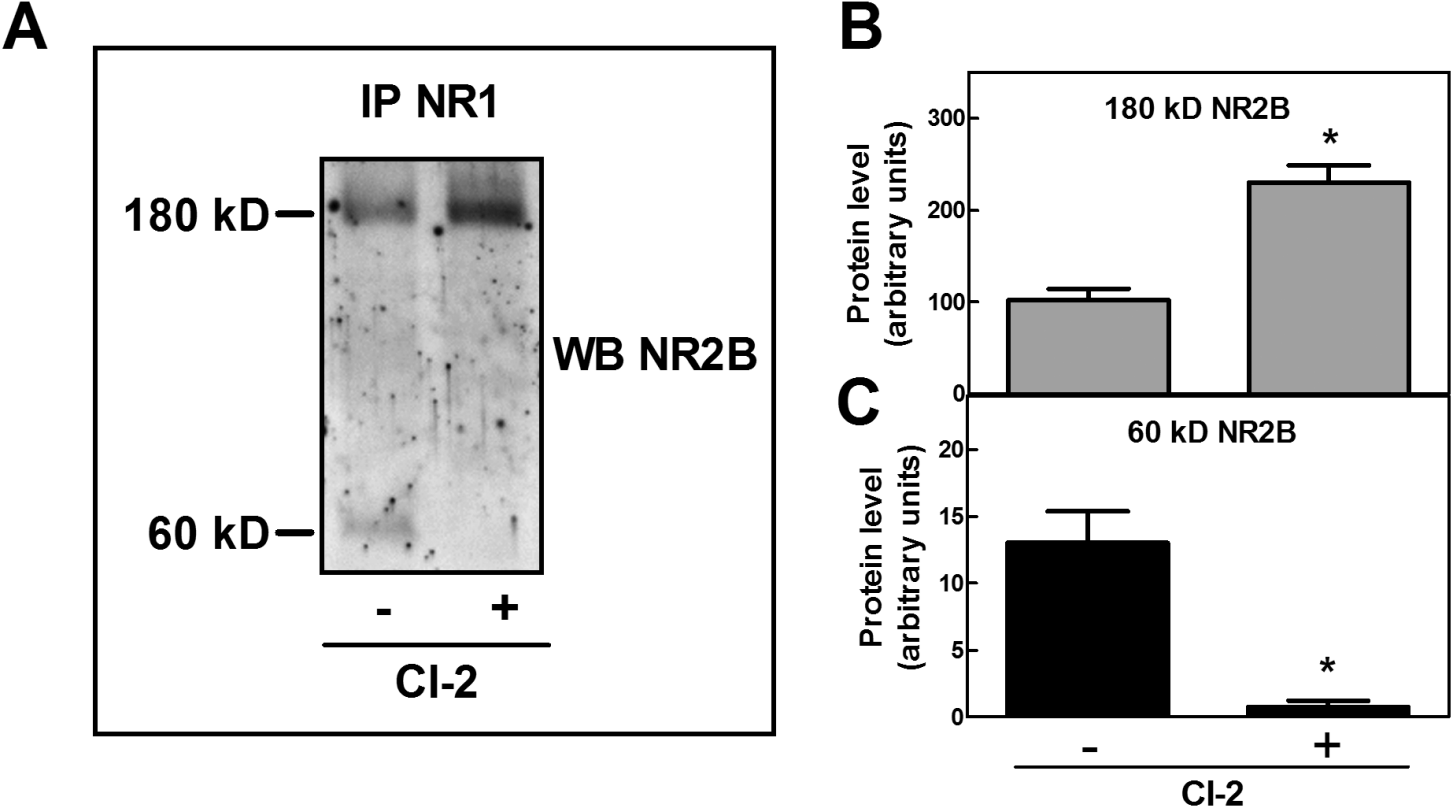
Effect of calpain inhibition on NR2B protein levels in SKNBE cells. (A) SKNBE cells were incubated for 24 hours in the absence or presence of 1 μM Calpain inhibitor 2 (CI-2), and lysed to perform immunoprecipitation with 1 μg of anti-NR1 antibody. The immunoprecipitated material (IP NR-1) was analyzed by immunoblotting for NR2B (WB NR2B). (B and C) The protein bands detected in (A) were quantified as described in Methods. Each value represents the arithmetical mean ± SEM of four different experiments. * *p* < 0.01 vs control (- CI-2), according to *t*-test.

To characterize the role of calpain in the constitutive digestion of NR2B, SKNBE cells were cultured for 24 hours in the presence of the synthetic calpain inhibitor CI-2 and the levels of NR2B species were measured. As shown in Fig 3A, 3B and 3C untreated SKNBE cells contain, in addition to the native 180 kD NR2B, the 60 kD fragment in amounts ranging from 10 to 15% of total. Instead, cell exposure to CI-2 for 24 hours induced a 2–3-fold increase in the level of native 180 kD NR2B and the complete disappearance of NR2B 60 kD fragment. Our observations demonstrate the direct involvement of the resident calpain 1 in the selective cleavage of NR2B subunit in basal conditions.

**Fig 4:**
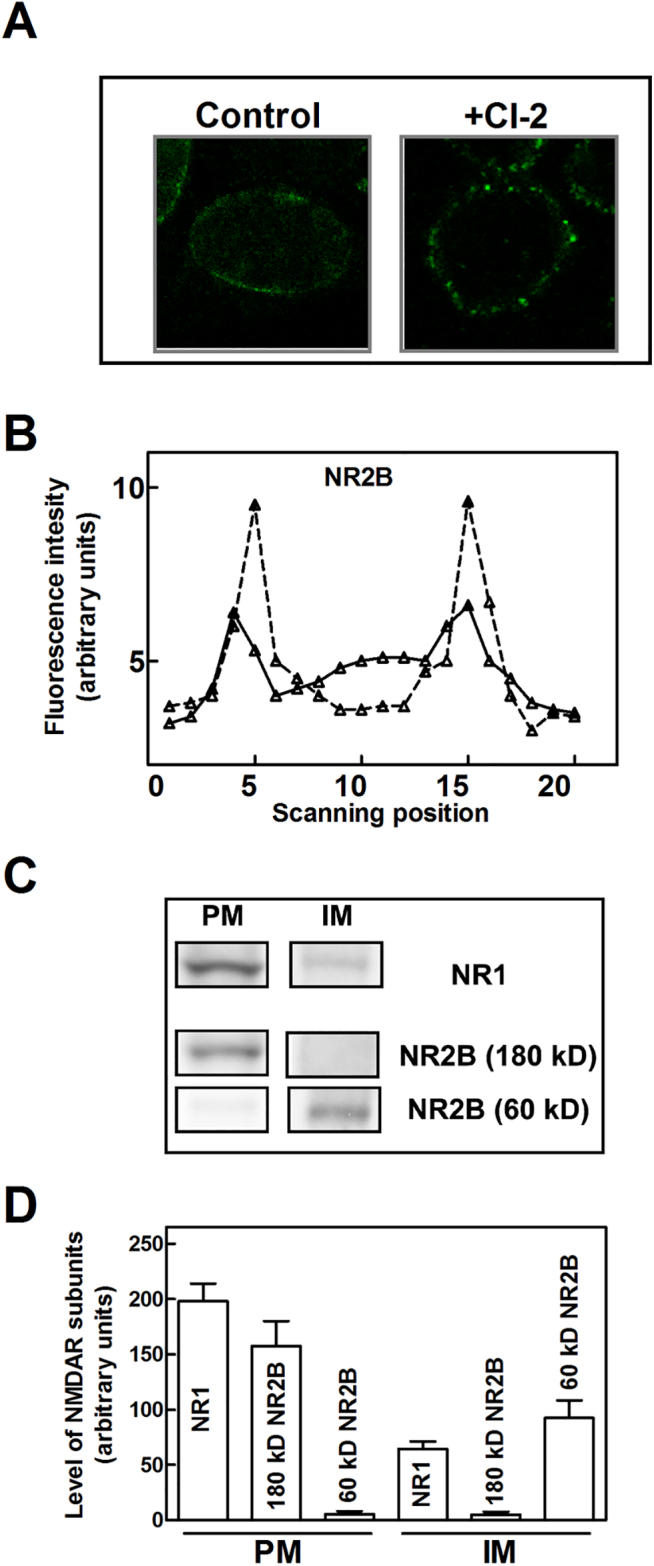
Effect of calpain inhibition on intracellular localization of NMDAR. (A and B) SKNBE cells untreated (Control, solid line) or treated with 1 μM CI-2 (+CI-2, dashed line) for 24 hours were fixed and NR2B localization was determined by confocal microscopy (see Methods). NR2B signal (green fluorescence) was continuously monitored during cell scanning by using Laser Pix software, as previously reported [54]. Each scanning trail is representative of 20 cells analyzed. (C) Aliquots (100 μL) of cell membranes were collected from the two fractions layered at 10% and 30% sucrose interface (see Methods) and assayed for 5′-nucleotidase activity, in order to determine the fraction containing the plasma membranes (PM) and the one containing internal membranes (IM). The same samples were analyzed by immunoblotting to detect the indicated proteins. (D) The protein bands detected in (C) were quantified as described in Methods. Each value represents the arithmetical mean ± SEM of three different experiments.

Inspection by confocal microscopy (Fig 4A) revealed that in resting cells 80–90% of NR2B subunits are localized at the plasma membranes and only 10–20% in the cytoplasm. When the cells were exposed to CI-2, the NR2B subunits were detected almost entirely at the plasma membranes and their level was 2−3-fold increased, as confirmed also by western blot analysis (see Fig 3). Concomitantly, the cytoplasmic fluorescence disappeared almost completely. These findings are suggesting that the accumulation of the native and the 60 kD NR2B species take place at different cell localization. To verify this hypothesis plasma membranes were separated from internal membranes by a sucrose gradient. As shown in Fig 4C and 4D, NMDAR containing NR1 and native NR2B subunits was detectable at the plasma membranes (PM) fraction, whereas NMDAR containing NR1 and digested NR2B subunits was present only in the internal membranes (IM) fraction. These data indicate that NMDAR containing the digested NR2B is rapidly removed from the plasma membrane and internalized in endosomes. Accordingly, it can be assumed that in basal conditions, calpain 1 plays an important role in regulating the level of functional NMDAR through the cleavage of NR2B at the C-terminal region thus favoring the formation of vesicles and the internalization of the receptor.

### Resident calpain 1 plays a role in a cell defense mechanism mediated by NMDAR internalization

**Fig 5:**
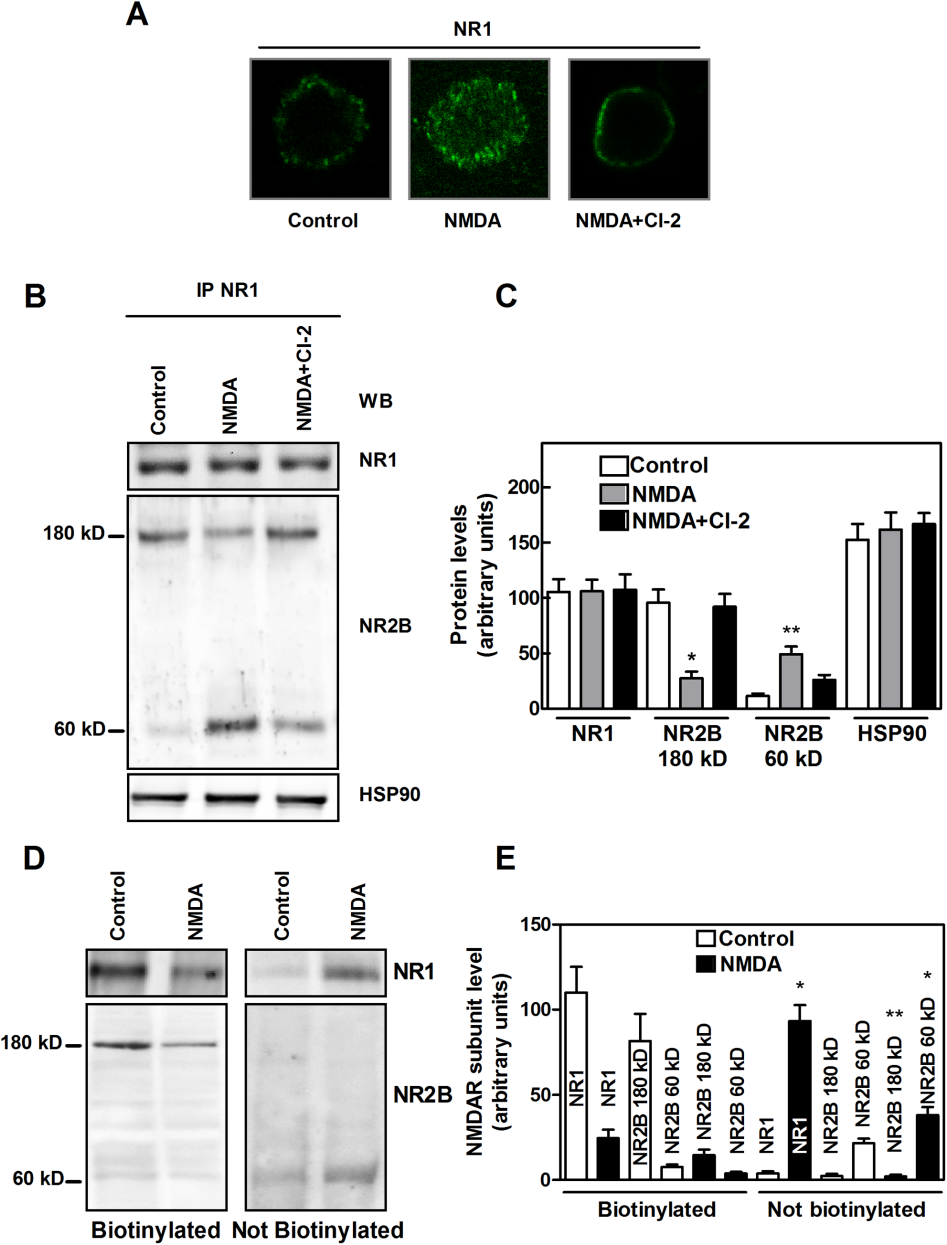
NMDAR translocation in SKNBE cells following activation of resident calpain 1. (A and B) SKNBE cells were incubated for 2 hours with 100 μM NMDA and 10 μM glicine in the absence (NMDA) or presence (NMDA+CI-2) of 1 μM CI-2, or left untreated (Control). The cells were then fixed and NR1 subunit localization (green fluorescence) was determined by confocal microscopy. Confocal microscopy micrographs are representative of four different experiments. Alternatively, after the indicated treatments, cells were then lysed to perform immunoprecipitation with 1 μg of anti-NR1 antibody and the immunoprecipitated material (IP NR1) was analyzed by immunoblotting (WB) to detect the indicated proteins. (C) The protein bands detected in (B) were quantified as described in Methods. Each value represents the arithmetical mean ± SEM of three different experiments. * *p* < 0.01 vs control and NMDA+CI-2; ** *p* < 0.01 vs control and *p* < 0.05 vs NMDA+CI-2, according to ANOVA followed by post-hoc Tukey’s test. (D) Cell surface expression of NR1 and NR2B subunits was also evaluated by biotinylation assay (see Methods) after incubation of SKNBE cells in the absence (Control) or presence (NMDA) of 100 μM NMDA for 2 hours. NR1 and NR2B subunits were detected by immunoblotting on a fraction (30 μL) of eluted (Biotinylated) or unbound (Not Biotinylated) proteins. (E) The protein bands detected in (D) were quantified as described in Methods. Each value represents the arithmetical mean ± SEM of three different experiments. * *p* < 0.001 and ** *p* < 0.05 vs the relevant protein in biotinylated material following cell stimulation, according to *t*-test.

To further explore the effect of resident calpain 1 on the properties of NMDAR complex, SKNBE cells were exposed to 100 μM NMDA to induce a Ca^2+^ influx across the receptor channel, a condition that, as previously indicated, activates only resident calpain 1 without affecting cell viability. Confocal microscopy images revealed that NMDAR containing native NR1 subunits was internalized into cytoplasmic vesicles (Fig 5A). Since this process was prevented by the addition of CI-2, we can confirm that resident calpain 1 is directly involved in inducing NMDAR internalization. In NMDA-stimulated cells, only NR2B subunits were extensively digested, whereas both NR1 and HSP90 were not affected (Fig 5B and 5C). As expected, CI-2 prevented NR2B proteolysis.

The expression of NMDAR on the plasma membranes of SKNBE cells was also analyzed by biotinylation experiments. As shown in Fig 5D and 5E, we observed that NR1, resistant to calpain proteolysis, disappears from cell surface in parallel with the digestion of NR2B. These data demonstrate that digestion of NR2B is critical for the internalization of NMDAR. This event could be indicative of a specific physiological function of resident calpain 1 operating in the regulation of the amount of NMDAR molecules present at the plasma membranes and thus of the intensity of Ca^2+^ influx through the receptor channel.

### Calpain 1 activity in NMDAR cluster is controlled by HSP90

**Fig 6:**
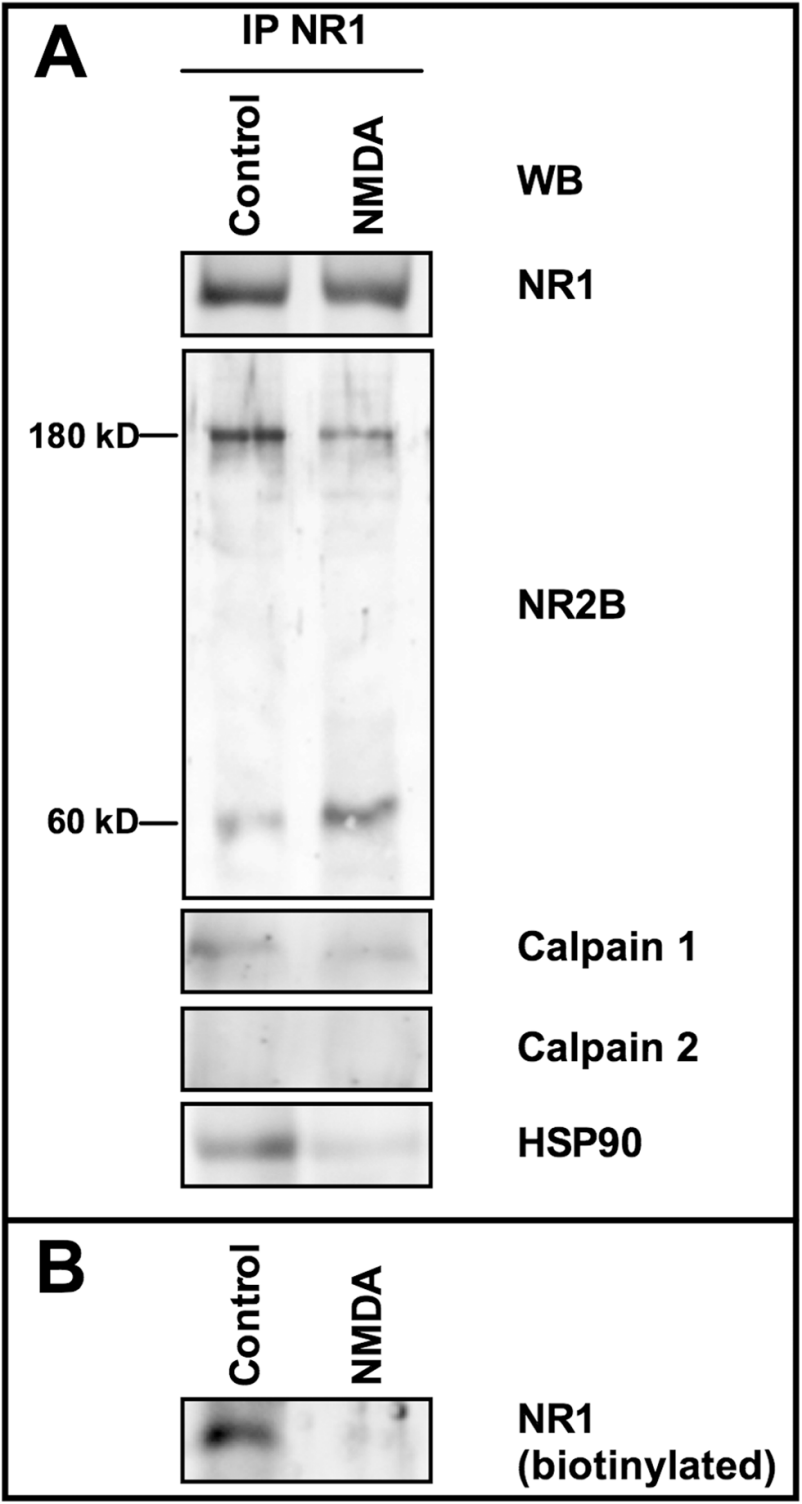
NMDAR cluster modifications in rat hippocampal synaptosomes following calpain activation. (A) Rat hippocampal synaptosomes were incubated at 37°C for 12 min with (NMDA) or without (Control) 100 μM NMDA in HEPES buffer containing 1 μM glycine and 1 mM CaCl_2_. Synaptosomes were lysed and immunoprecipitation was carried out with 1 μg of anti-NR1 antibody. The immunoprecipitated material (IP NR1) was analyzed by immunoblotting (WB) to detect the indicated proteins. (B) Surface localization of NR1 subunit was also evaluated by biotinylation assay in rat hippocampal synaptosomes treated as in (A). NR1 was detected on a fraction (30 μL) of eluted proteins (biotinylated) by immunoblotting. Each immunoblot is representative of four different experiments.

To obtain more information on calpain-mediated proteolysis/internalization of NMDAR, we have investigated on the NR1/NR2B receptor present in hippocampal synaptosomes purified from rat nerve terminals. As shown in Fig 6A, in total synaptosome preparation the NR1-immunoprecipitated multiprotein complex contained calpain 1, and neither calpain 2 nor calpastatin. Moreover, HSP90 was present together with native NR2B, and small amounts of the 60 kD NR2B fragment. Thus, calpain 1 results to be constitutively associated also to the NMDAR localized in synaptosomes. Presumably the presence of the 60 kD NR2B fragment in unstimulated synaptosomes is the result of a calpain-mediated digestion of native NR2B. Indeed, when synaptosomes were exposed to 100 μM NMDA, the concentration inducing the maximal efflux of glutamate [35], a remarkable disappearance of the native NR2B subunit occurred, together with the accumulation of the 60 kD NR2B digested form (Fig 6A). In addition, the amount of resident calpain 1 did not increase following NMDA stimulation, and neither calpain 2 nor calpastatin were recruited in the NMDAR complex. Moreover, in these structures, HSP90 was extensively digested and NR2B degradation corresponded to the removal of NMDAR from the plasma membranes, as indicated by the disappearance of the biotinylated native NR1 subunit (Fig 6B). Thus, the NR1/NR2B-containing NMDAR cluster, present in SKNBE cells and in synaptosomes, contains a resident calpain 1 that is partially active in basal conditions and becomes highly activated following stimulation of NMDAR.

**Fig 7:**
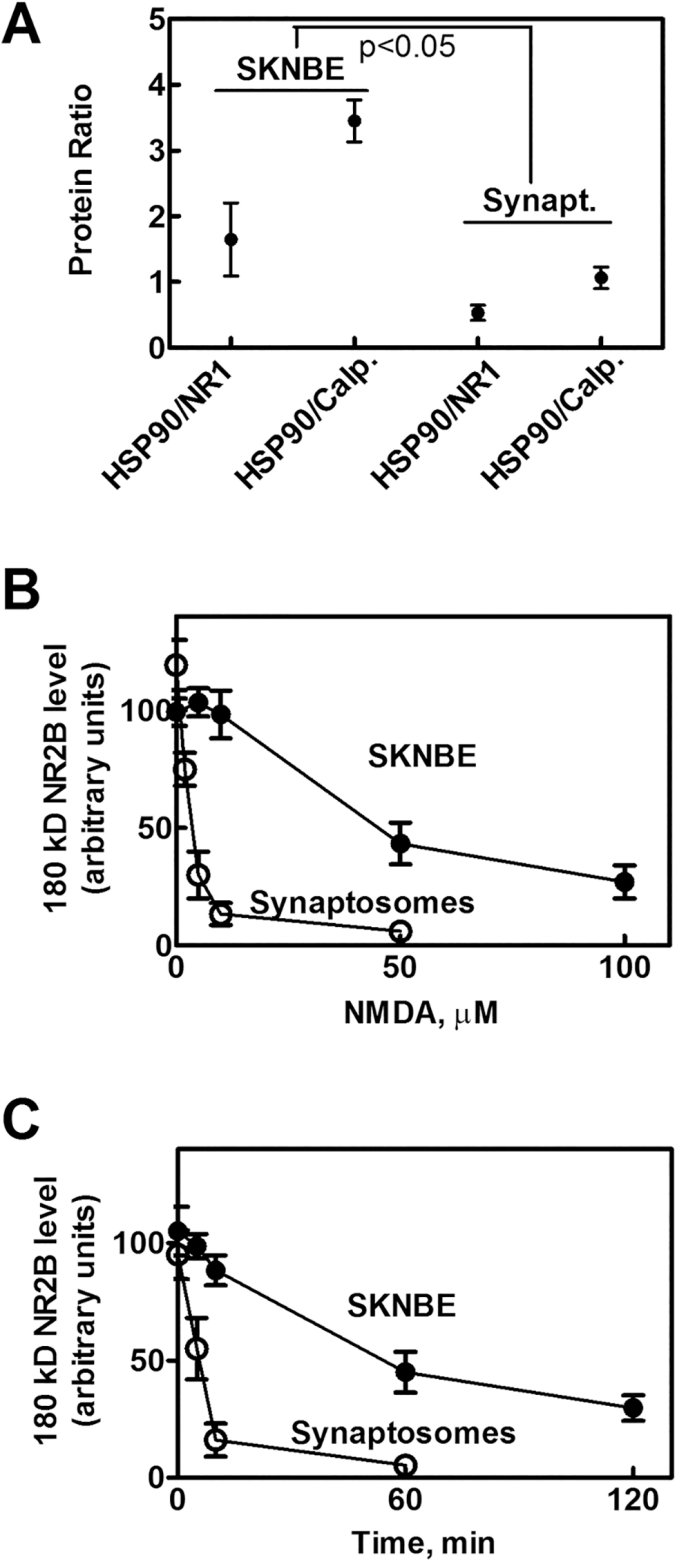
HSP90 levels and 180 kD NR2B degradation in rat hippocampal synaptosomes. (A) The immunoreactive bands (corresponding to HSP90, NR1 subunit, and Calpain 1) obtained by immunoprecipitation from SKNBE cells (see Fig 1A and relevant legend) and rat hippocampal synaptosomes (see Fig 6A and relevant legend) were quantified and the indicated protein ratio were calculated. The values reported are the arithmetical mean ± SEM of four different experiments. * *p* < 0.05, according to ANOVA followed by post-hoc Tukey’s test. (B) Quantification of the immunoreactive bands corresponding to 180 kD NR2B subunit immunoprecipitated from the indicated samples incubated for 2 hours at different concentrations of NMDA. (C) Quantification of the immunoreactive bands corresponding to 180 kD NR2B subunit immunoprecipitated from the indicated samples incubated with 100 μM NMDA at different times. The values reported are the arithmetical mean ± SEM of three different experiments.

Taken together these findings indicate that the NMDAR complexes in SKNBE cells and hippocampal synaptosomes contain the same components although in different ratios (Fig 7A). Specifically, in the NMDAR cluster from synaptosomes, the HSP90/calpain ratio is 3−4-fold lower than in unstimulated SKNBE cells. As a consequence, calpain 1 resident in the NMDAR of synaptosomes appears less efficiently controlled. The lack of regulation by HSP90 is further confirmed by the fact that in synaptosomes, unlike in SKNBE cells, NR2B is nearly completely degraded already at 10 μM NMDA (Fig 7B), and the rate of this digestion is about 10–fold faster than that observed in SKNBE cells (Fig 7C).

**Fig 8:**
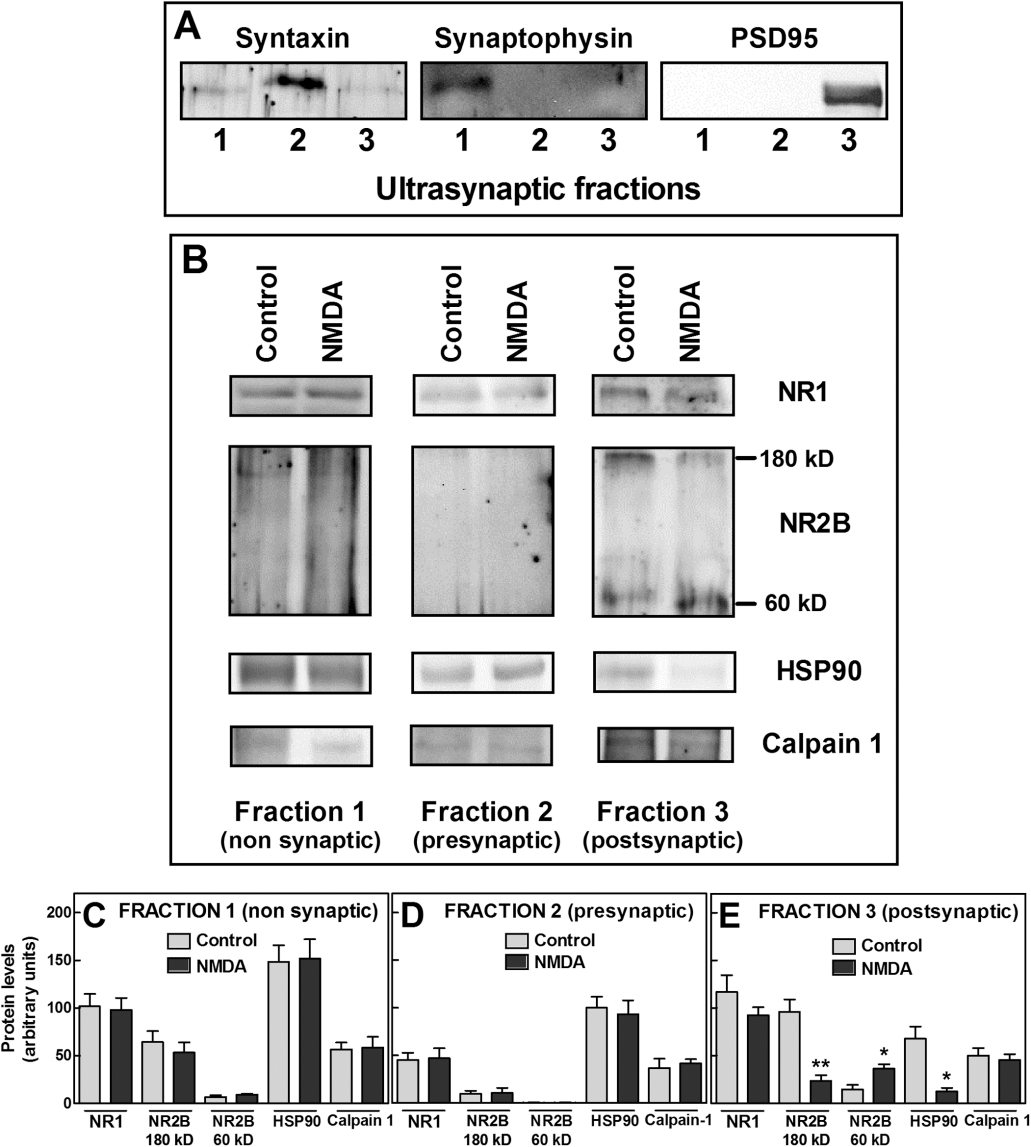
NMDAR cluster modifications in ultrasynaptic fractions from rat hippocampal synaptosomes. Rat hippocampal synaptosomes were incubated at 37°C for 12 min with (NMDA) or without (Control) 100 μM NMDA in HEPES buffer containing 1 μM glycine and 1 mM CaCl_2_. Synaptosomes were then lysed and ultrasynaptic fractionation was carried out as described in Methods. (A) Aliquots (20 μg according to Lowry assay) of each fraction were submitted to 10% SDS-PAGE followed by immunoblot for the indicated markers. Lane 1, non synaptic fraction; lane 2, presynaptic fraction; lane 3, postsynaptic fraction. A representative immunoblot (of three) is shown. (B) Immunoprecipitation was carried out on each fraction by using anti-NR1 antibody. The immunoprecipitated material was analyzed by immunoblotting to detect the indicated proteins. Each immunoblot is representative of three different experiments.(C, D, and E) The protein bands detected in (B) were quantified as described in Methods. Each value represents the arithmetical mean ± SEM of three different experiments. ** *p* < 0.01 and * *p* < 0.05 vs control, according to *t*-test.

These results confirm that also in synaptosomes, resident calpain 1 is involved in controlling the amount of NMDAR at the plasma membranes and that the protease activity, and thus the extent of NR2B digestion, is in turn controlled by the amount of HSP90 associated to the protein cluster. Since the synaptosome preparation used in these experiments contains pre-, post- and non synaptic elements, we have separated these different compartments [49], and as shown in Fig 8A, investigated the distribution of well characterized marker proteins (PSD95, syntaxin and synaptophysin) in the purified fractions. Consistent with the previous report [49], PSD95 was predominant in the postsynaptic fraction, syntaxin was highly enriched in the presynaptic fraction and synaptophysin was particularly abundant in the non synaptic fraction. In all the fractions, in addition to NR1, both calpain 1 and HSP90 although at different levels (Fig 8B) were also present. As shown in Fig 8E, we observed that the digestion of NR2B was more intense in the postsynaptic fraction and it was accompanied by degradation of HSP90. On the contrary, in the non synaptic fraction, proteolysis of both native NR2B and HSP90 was not appreciable (Fig 8C). Instead, in the presynaptic fraction, NR2B was poorly detectable (Fig 8D) even if HSP90 was highly represented and in large excess in respect to calpain 1. It is interesting to note that, as shown in Table 1, the susceptibility to digestion of NR2B is correlated to the level of HSP90 associated to the NMDAR complex. In particular, in the postsynaptic compartment, in which we have observed a low HSP90/calpain 1 ratio, the protease resulted more easily activable promoting presumably the internalization of NR1/NR2B-containing NMDAR, as observed in synaptosomes.

**Table 1:** NR2B susceptibility to digestion by resident calpain 1 as a function of HSP90 level.

**Source**	**HSP90/calpain 1 ratio**	**180 kD NR2B level (% of control)**
SKNBE cells	3.45 ± 0.33	86.5 ± 12.5
Synaptosomes	1.06 ± 0.17	19.1 ± 10.2
Non synaptic fraction	2.80 ± 0.66	91.8 ± 26.2
Postsynaptic fraction	1.36 ± 0.22	26.2 ± 9.8

HSP90/calpain 1 ratio values were extrapolated from Fig 7A (for SKNBE cells and synaptosomes), and from Fig 8C and 8E (for non synaptic and postsynaptic fractions, respectively). Native NR2B levels were extrapolated from Fig 7C (for SKNBE cells and synaptosomes), and from Fig 8C and 8E (for non synaptic and postsynaptic fractions, respectively), and were calculated as percentage of control following 12 min-exposure to NMDA. Data are presented as the arithmetical mean ± SEM.

## Discussion

It is well known that Ca^2+^ entry through NMDAR, not only mediates various neuronal activities and signaling pathways, but also induces calpain activation that in turn catalyzes the selective processing of the receptor [4, 8, 11, 16, 51, 52].

We have reported [34] that in SKNBE cells saturating amounts of calpain 1 are stably associated to NR1/NR2B-containing NMDAR multiprotein complex. In the present study, we have explored the mechanisms underlying the activation of such resident calpain and the biological significance of this specific proteolytic activity.

The amount of calpain 1 at the NMDAR complex is maximal in resting conditions and does not change following NMDA stimulation. The specific localization of calpain 1 at the NMDAR confers to the protease both selectivity and “in situ” specificity, as calpain 2 was never detected at this site. Resident calpain 1 expresses in basal conditions a limited but detectable proteolytic activity capable to induce the selective cleavage of NR2B, followed by internalization of the NMDAR containing this digested subunit. Following stimulation with NMDA, the rate of NR2B digestion increases and as a consequence NMDAR is almost completely internalized.

These observations suggest that resident calpain 1 plays an important physiological role in the modulation of the NMDAR level and function at the plasma membranes. This role of calpain is confirmed by experiments involving cell exposure to a synthetic calpain inhibitor. In fact, the reduced activity of resident calpain results in increased amounts of native NR2B subunit, and thus of NMDAR, at the plasma membranes.

In this context, particularly important is the presence of HSP90 associated to calpain 1 [34] at the level of the NMDAR cluster where it can play several functions. Specifically, we have previously observed that HSP90 protects neuronal NO synthase from calpain-mediated digestion [53], and regulates the activity of the protease by reducing the affinity for calcium of calpain 1 [34]. The latter may have great physiological importance since calpain 1, once inserted in the NMDAR cluster, escapes from its natural inhibitor calpastatin [34]. Thus, HSP90 becomes the alternative regulatory agent for calpain activity in this specific localization. Although HSP90 is also a calpain substrate, digestion occurs exclusively on the free chaperone molecules and not on those already associated to the protease. In this way, the regulatory function of HSP90 is preserved. Our data suggest that the activity of calpain resident at the NMDAR cluster is directly dependent on the amount of HSP90 associated to the protease.

We have observed that also in synaptosomes, calpain 1 is constitutively associated to the NR1/NR2B complex in an active form, as indicated by the presence of the 60 kD NR2B species. However, to promote the complete degradation of the native NR2B subunit, it is sufficient a concentration of NMDA 10-fold lower than that used in SKNBE cells. The higher calpain activation occurring in the NMDAR cluster of synaptosomes could be explained by lower amounts of HSP90 associated to resident calpain 1 than those present in SKNBE cells. Such low levels of HSP90 are, following stimulation with the agonist, ultimately responsible for the very rapid digestion of NR2B and the consequent NMDAR internalization. In this condition we also observe the complete disappearance of the chaperone. The role of HSP90 in the regulation of the digestion of the NMDAR is further confirmed by the characterization of the reciprocal ratio of specific proteins associated to the NR1/NR2B complex in the isolated ultrasynaptic compartments. Particularly, we have observed that in the postsynaptic fraction NMDAR, containing a reduced amount of HSP90, is more susceptible to calpain-mediated proteolysis as compared to the non synaptic NMDAR cluster that, containing higher levels of HSP90, results less susceptible to proteolysis.

In conclusion, we are here demonstrating that differences in the properties of the NMDAR cluster, besides different localization, could be also due to the content of specific proteins, and particularly of HSP90 and calpain 1. A higher rate of calpain 1 activation, as well as the internalization of NMDAR, could be visualized either as a mechanism to promote cell responses rapidly operating or to induce cell protection against excitotoxic ion influx. Anyway, the calpain 1 activity detectable in basal conditions is involved also in the physiological maintenance of optimal levels of NMDAR at the plasma membranes.

## Supporting Information

S1 Fig SKNBE cell viability following exposure to NMDA. SKNBE cells were incubated for 24 hours with 100 μM NMDA and 10 μM glicine in the absence (NMDA) or presence (NMDA+CI-2) of 1 μM calpain inhibitor 2 (CI-2), or left untreated (Control). Cell viability was evaluated by means of the neutral red uptake as described in Methods. Values are reported as percentage of control and are presented as mean ± SEM of three different experiments.
(TIF)

S1 File Minimal data set. (XLS)
