# Supplementary figures and images for: Physiological Roles of Calpain 1 Associated to Multiprotein NMDA Receptor Complex
Source: PLoS One. 2015 Oct 2;10(10):e0139750. doi: 10.1371/journal.pone.0139750 (PMC4592069; doi:10.1371/journal.pone.0139750)

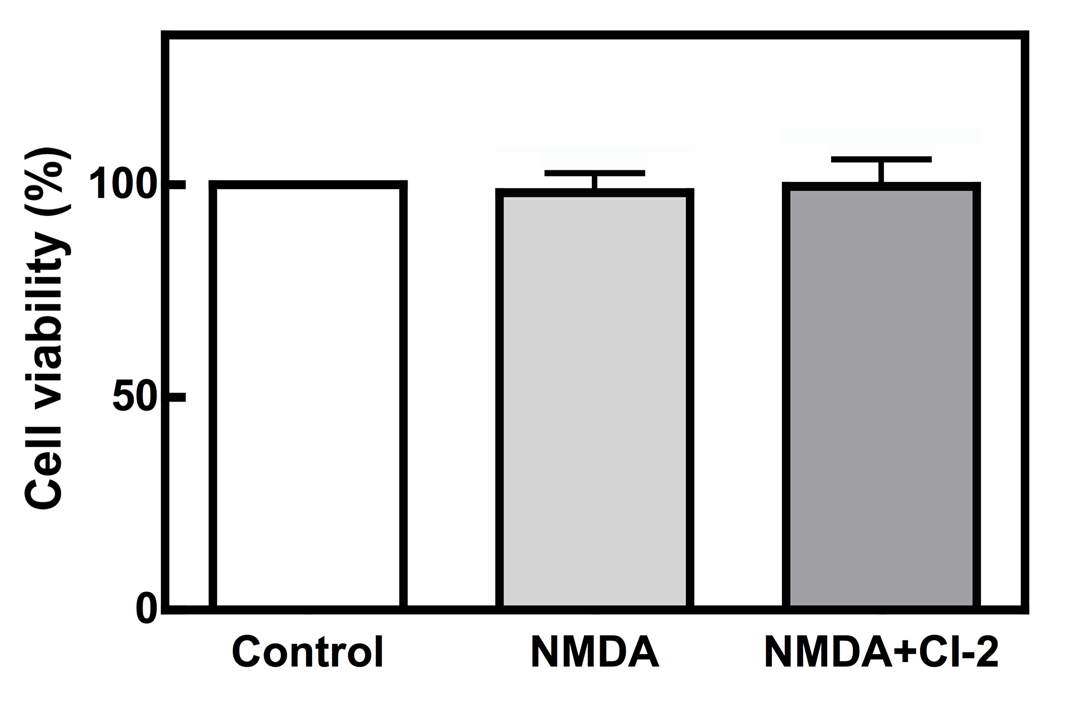

Supplement: S1 Fig — SKNBE cells were incubated for 24 hours with 100 μM NMDA and 10 μM glicine in the absence (NMDA) or presence (NMDA+CI-2) of 1 μM calpain inhibitor 2 (CI-2), or left untreated (Control). Cell viability was evaluated by means of the neutral red uptake as described in Methods. Values are reported as percentage of control and are presented as mean ± SEM of three different experiments. (TIF) [file pone.0139750.s001.tif]
